# Discovery of novel *Schistosoma japonicum* antigens using a targeted protein microarray approach

**DOI:** 10.1186/1756-3305-7-290

**Published:** 2014-06-25

**Authors:** Hamish EG McWilliam, Patrick Driguez, David Piedrafita, Donald P McManus, Els NT Meeusen

**Affiliations:** 1 Department of Microbiology and Immunology, The University of Melbourne, at the Peter Doherty Institute for Infection and Immunity, Melbourne, Victoria, Australia; 2 Molecular Parasitology Laboratory, QIMR Berghofer Medical Research Institute, Brisbane, Queensland, Australia; 3 School of Applied Sciences & Engineering, Federation University, Churchill, Victoria, Australia; 4 Department of Microbiology, School of Biomedical Sciences, Monash University, Melbourne, Victoria, Australia

**Keywords:** *Schistosoma japonicum*, Vaccine development, Ly-6 proteins, Protein microarray, Immunomics

## Abstract

**Background:**

Novel vaccine candidates against *Schistosoma japonicum* are required, and antigens present in the vulnerable larval developmental stage are attractive targets. Post-genomic technologies are now available which can contribute to such antigen discovery.

**Methods:**

A schistosome-specific protein microarray was probed using the local antibody response against migrating larvae. Antigens were assessed for their novelty and predicted larval expression and host-exposed features. One antigen was further characterised and its sequence and structure were analysed *in silico*. Real-time polymerase chain reaction was used to analyse transcript expression throughout development, and immunoblotting and enzyme-linked immunosorbent assays employed to determine antigen recognition by antibody samples.

**Results:**

Several known and novel antigens were discovered, two of which showed up-regulated transcription in schistosomula. One novel antigen, termed *S. japonicum* Ly-6-like protein 1 (Sj-L6L-1), was further characterised and shown to share structural and sequence features with the Ly-6 protein family. It was found to be present in the worm tegument and expressed in both the larval and adult worms, but was found to be antigenic only in the lungs that the larvae migrate to and traverse.

**Conclusions:**

This study represents a novel approach to vaccine antigen discovery and may contribute to schistosome vaccine development against this important group of human and veterinary pathogens.

## Background

An effective vaccine against schistosomiasis would be a major step towards eliminating this devastating and widespread tropical parasitic disease, and has been the focus of significant research effort for decades [1]. Numerous schistosome vaccine candidates have been discovered showing varying levels of protection, and several molecules are in development [2]; however, a commercial vaccine still remains elusive. It has been suggested that more effective vaccine candidates remain undiscovered, indicating a need for novel approaches to target discovery [1,3].

The radiation-attenuated larval vaccine model provides evidence that high levels of protection can be generated against schistosome infection. When a range of animal models are infected with radiation-attenuated cercariae, the resulting immunity rejects up to 90*%* of the challenge infection [4,5]. In this model the larvae do not progress to adult worms, and the consensus is that the early developing schistosomula represent a potent source of protective antigens and are the target of this protective immune response [4,5]. Furthermore, larval stages are also suspected of being the target of naturally acquired immunity in humans [6-8]. In contrast, the adult worms can live in the host bloodstream for decades despite being surrounded by specific components of the immune response [9,10]. Therefore larval-specific antigens, or molecules exposed uniquely in larvae, have the potential to serve as effective novel vaccine candidates. Despite this, the current schistosome vaccine targets are largely adult antigens and there is a lack of larval-specific antigens being evaluated [11].

Recently the genomes of all three major schistosome species have been sequenced [12-14], and several post-genomic approaches and high throughput methods have been developed to take advantage of this wealth of information [15]. One such approach is a schistosome-specific protein microarray, which contains 232 unique antigens displayed on nitrocellulose slides [16]. These proteins were selected from bioinformatic data using criteria biased towards promising vaccine candidates i.e. up-regulated expression in larvae, predicted/known parasite surface expression, and limited similarity with mammalian sequences [16]. Many of these are novel molecules and the majority are from *Schistosoma japonicum*, with the remainder from *S. mansoni*. The arrayed proteins can be probed with antibodies from immune hosts as a powerful new technology for vaccine antigen discovery [11].

In the present study, this protein microarray was screened with antibodies generated from lymph nodes draining the sites of larval *S. japonicum* migration [17]. Referred to as antibody secreting cell-probes (ASC-probes), these antibodies are specific to the migrating skin and lung larvae [17], and were generated from *S. japonicum*-infected rats, which is a well-studied model of antibody-mediated immunity against the migrating larvae during a secondary infection [18-20]. Hence, these ASC-probes have the potential to recognise protective larval antigens. Using this approach we identified several novel antigens expressed by schistosomula; one promising candidate was further investigated indicating it may be an important target for vaccine development.

## Methods

### Ethics statement

The conducts and procedures involving animal experiments were approved by the Monash University animal ethics review board, and the Animals Ethics Committee of the Queensland Institute of Medical Research (project no. P288). This study was performed in accordance with the recommendations of the Australian code of practice for the care and use of animals for scientific purposes, 2004.

### Protein microarray screening

A protein microarray consisting of 232 unique schistosome proteins was prepared as described by Driguez et al. [16]. The microarray slides were hydrated using Blocking Buffer (BB; Whatman) for 30–60 min at RT, using 16-pad incubation chambers and frames (Whatman) to separate arrays. Antibodies used for screening the microarrays were obtained from lymph nodes of challenged rats as detailed in McWilliam et al. [17]. Briefly, previously infected rats were challenged with 350 cercariae and skin and lung lymph nodes (LN) removed at 5 and 9 days after challenge respectively. This protocol was designed based on previous observations that rats show antibody-mediated immunity against larvae during secondary infections [18,19]. LN cell suspensions were cultured *in vitro* for 5 days to allow spontaneous secretion of antibodies by *in vivo* induced antibody secreting cells (ASC) and the supernatant collected and used as the antibody probe (=ASC-probe). Considering ASC-probes from non-infected (NI) rats contain little antibody [16], they are not ideal controls for non-specific antibody binding; NI rat sera was therefore used as the negative control. Four skin and four lung ASC-probes (undiluted) or 15 NI serum samples (1:100 in BB) were incubated on the arrays in a sealed box overnight at 4°C. Skin and lung ASC-probes were selected based on those containing the highest antibody concentration as documented in [16]. Arrays were washed 3 times with tris-buffered saline (TBS; 20 mM tris, 150 mM NaCl) with 0.05% Tween-20 (TBST), followed by biotin-conjugated anti-rat IgG (1:1000 in BB) for 1 h at RT. After 3 washes in TBST, streptavidin-conjugated Cy5 fluorophore (Surelight P3, Columbia Biosciences; 1:200 in BB) was incubated for 1 h at RT. After a final 3 washes in TBST the array slides were separated from the chambers, washed in distilled water and dried by centrifuging for 5 minutes at 500 × *g*. The slides were stored in the dark until scanned.

### Microarray scanning and antigen identification

Scanning was performed on a confocal laser microarray scanner (Genepix 4300A, Molecular Devices), and the signal intensity (SI) was quantified using image analysis software (Genepix Pro 7, Molecular Devices) and transformed and normalised using the vsn statistical package (http://www.r-project.org). Finally the data were re-transformed (inverse log_2_) to a normalised SI [21-23].

Antigens were considered positively recognised by an ASC-probe sample when the SI was greater than two standard deviations above the mean of NI rat sera. Amino acid sequences of the identified antigens were then analysed for features of potentially important vaccine candidates i.e. developmental expression based on EST Profile Viewer (http://www.ncbi.nlm.nih.gov/ unigene), presence of a signal peptide using SignalP -4.1 (http://www.cbs.dtu.dk/services/SignalP), and a predicted transmembrane domain with TMpred (http://www.ch.embnet.org/software/TMPRED_form.html). Also, literature searches were performed to determine the novelty of each antigen, which were considered novel when there were no reports of vaccine efficacy against *S. japonicum* or significant characterisation.

### RNA isolation from schistosome life-stages

Cercariae were obtained from freshly-shed infected *Oncomelania hupensis hupensis* snails and transferred using a sterile bacterial loop directly to Qiazol Lysis Reagent (Qiagen). Two-day (2d) schistosomula were manually transformed from cercariae using the syringe method as described in McWilliam et al. [17], and cultured *in vitro* for 2 days at 37°C with 5% CO_2_. Three-day (3d) schistosomula were obtained from infected mice lungs 3 days post-infection (lung-stage schistosomula) as described by Gobert et al. [24], and were lysed directly in Qiazol. Adult worm pairs were obtained from freshly-perfused mice, and either left as pairs or carefully separated, and washed in PBS before homogenising in Qiazol. The RNA was then extracted from each stage following the manufacturer’s protocol and stored in water at -80°C until required.

### Developmental expression of novel antigens by quantitative real-time PCR (qPCR)

After determining the RNA concentration using a NanoDrop spectrophotometer (Thermo Scientific), total RNA (300 ng) was converted to cDNA using the QuantiTect Reverse Transcription Kit (Qiagen) including the genomic DNA removal step. To perform the qPCR, primers were designed based on the novel antigen sequences identified in the protein microarray screening and using NADH dehydrogenase ubiquinone flavoprotein 2 (NDUFV2) as the reference gene [25]. The primer sequences were: AY815838 (forward: 5′- CGTCGACATTCAAGTTGGTC -3′, reverse: 5′- GGGGCATAATCTTCACTTTGA -3′); Ly-6-like-1 (forward: 5′- TGAAAGTTTTGGGACTTTGTATG -3′, reverse: 5′- CGAATGGATTCGGACAGTCT -3′); calponin-like (forward: 5′- CATGTCATTCGGTGCTCAAC -3′, reverse: 5′- TTCAGCAATATGACGTTGATTACTA -3′) and NDUFV2 (forward: 5′- CGAGGACCTAACAGCAGAGG -3′, reverse: 5′- TCCGAACGAACTTTGAATCC -3′). Because calponin-like protein has homology to another calponin homologue in *S. japonicum* (accession #AAD11976), the primers were designed in the most dissimilar region. The qPCR reactions included SYBR Master Mix (Applied Biosystems) and the above primers at 0.5 *μ*M and 5 *μ*l of 1:20 diluted cDNA, and were run in technical triplicates on an Eppendorf Realplex4 Mastercycler for 35 cycles, using an annealing temperature of 55°C. Melt curve analysis was performed to ensure a single product was amplified. The relative copy number of SYBR green for each gene was calculated from a standard curve of serial dilutions of cDNA, and the relative expression of the target gene was determined relative to the reference gene copy number. Finally, the expression of each gene was calculated relative to the cercarial cDNA level to observe the change throughout development.

### Sequence analysis of *S. japonicum* ly-6-like-1 (Sj-L6L-1)

The NCBI databases (http://blast.ncbi.nlm.nih.gov/Blast.cgi) were used to identify Sj-L6L-1 related *S. japonicum*, *S. mansoni*, and mammalian species (*Mus musculus* and *Homo sapiens*). For other parasitic trematodes (*S. haematobium*, *Fasciola hepatica*, *F. gigantica*, *Opisthorchis viverrini* and *Clonorchis sinensis*), searches were performed using the databases at http://www.gasserlab.org. Sequence alignments were performed by ClustalW2 (http://www.ebi.ac.uk/Tools/msa/clustalw2/). The presence of a signal peptide and omega site (which denotes the position where the C-terminal propeptide is cleaved off in the mature protein and is replaced with a glycosylphosphatidylinositol (GPI)-anchor) was predicted by SignalP -4.1 (http://www.cbs.dtu.dk/services/SignalP/) and big-PI predictor (http://mendel.imp.ac.at/gpi/gpi_server.html), respectively. Structural homology searches were performed using the amino acid sequence of mature Sj-L6L-1 without the predicted N-terminal signal peptide and C-terminal propeptide (beginning at M20 and truncated at the predicted GPI-anchor attachment site at N96) in the Phyre2 server (http://www.sbg.bio.ic.ac.uk/phyre2) [26], and the protein structure was displayed using PyMOL (v1.3; http://www.pymol.org).

### Production and purification of recombinant Sj-L6L-1 (rSj-L6L-1)

The Sj-L6L-1 sequence was amplified from the pXi T7 vector used in the construction of the protein arrays [16]. Primers were designed to produce the mature Sj-L6L-1 protein (M20-N96) (forward primer: 5′-ATGAAAAATAAAAAGGTCAAATG-3′, reverse: 5′-ATTACAATAATCTTCATCACAAC-3′). Amplification of the 231 bp Sj-L6L-1 fragment was performed using Phusion High Fidelity DNA Polymerase (New England Biolabs). Next 3′ adenine overhangs were added by incubating the PCR product with 1 unit of Platinum Taq DNA Polymerase (Life Technologies) and 0.2 mM dATP for 10 min at 72°C. This was purified and then cloned in-frame into the pBAD/TOPO ThioFusion plasmid (Life Technologies) according to the manufacturer’s directions. This resulted in a fusion protein with thioredoxin (*Escherichia coli*) as a solubility tag and 6His purification tag at the amino- and carboxy- termini, respectively.

The plasmid was sequenced to confirm the correct sequence, and then transformed into TOP10 *E. coli* cells (Life Technologies). Expression was induced by adding arabinose (0.005% w/v), and soluble rSj-L6L-1 present in the bacterial lysate supernatant was purified on a HisTrap HP column (GE Healthcare) according to the manufacturer’s instructions. Fractions were analysed by sodium dodecyl sulphate-polyacrylamide gel electrophoresis (SDS-PAGE) and the purest fraction was dialysed into TBS (pH 8.0) and stored at -20°C. The thioredoxin fusion partner alone (with the 6His tag; referred to as rTrx) was also produced using the ‘empty’ pBAD vector, and purified in the same way for use as a control.

Antiserum against rSj-L6L-1 and rTrx was generated by injecting a rat with 50 *μ*g of rSj-L6L-1 or rTrx with 200 μg Quil A in 0.1 ml PBS. A secondary immunisation was administered 2 weeks later, after which a test bleed indicated a specific antibody response to the administered antigen.

### Schistosome protein extracts

Crude worm extracts were prepared by collecting each developmental stage (as above), washing with TBS, and homogenising in 1% (w/v) SDS in TBS. Samples were centrifuged at 12,000 × g for 15 min at RT. The protein concentration of the supernatants was determined by BCA assay (Pierce, Rockford). To determine whether Sj-L6L-1 was soluble and present in the tegument, adult worms were separated into different fractions. Tegument was gently removed by a modified freeze/thaw/vortex technique [27,28]. Briefly, washed and snap-frozen worm pairs were thawed in TBS (pH 7.5) on ice for 5 min, followed by vortexing for 5 × 1 sec to allow gentle removal of tegument. The supernatant was removed and centrifuged at 13,000 × g for 10 min at 4°C. The tegument pellet was resuspended in lithium dodecyl sulphate (LDS) sample buffer (Life Technologies) and heated to 95°C for 10 min. The remaining worms were washed in 20 mM Tris (pH 7.4) and then homogenised in the same buffer. After centrifugation, as before, the supernatant was kept as the soluble fraction. The pellet was washed twice with buffer followed by another centrifugation step, and then solubilised in 1% SDS. After spinning again, the supernatant was kept as the aqueous-insoluble fraction.

### Western blotting for detection of native and recombinant Sj-L6L-1

Purified rSj-L6L-1 or rTrx (1 *μ*g) in LDS sample buffer with or without reducing agent (50 mM dithiothreitol; DTT), were run on 10% NuPAGE Bis-Tris gels, along with Novex Sharp Pre-stained Protein Standards (Life Technologies) and stained with Coomassie blue. For western blotting, rSj-L6L-1 (0.5 *μ*g) or worm extracts (10 *μ*g) were separated by SDS-PAGE and transferred onto nitrocellulose membranes, which were then blocked overnight at 4°C in 5% w/v skim milk powder in PBST (SM-PBST). After washing three times in PBST, the primary antibodies were added: rSj-L6L-1 was probed with neat pooled rat lung-LN ASC-probes from infected rats; worm extracts were probed with either anti-rSj-L6L-1 or anti-rTrx antiserum (1:500 in 1% SM-PBST). After washing, anti-rat Ig (H + L):HRP (1:1000) (Life Technologies) was incubated with the membranes for 1 h at RT, and the membranes re-washed. Finally ECL substrate (GE Healthcare) was applied and chemiluminescence detected on Super RX film (Fujifilm).

### Recognition of rSj-L6L-1 by enzyme-linked immunosorbant assay (ELISA)

An ELISA was used to quantify the antigenicity of rSj-L6L-1 during schistosomiasis, using the ASC-probes and serum samples from infected rats as described in McWilliam et al. [17]. Some samples (particularly NI serum) showed binding to the Trx fusion partner alone (data not shown); therefore binding was measured to both rSj-L6L-1 and rTrx. Wells were coated with recombinant protein (3 μg/ml) in carbonate coating buffer (pH 9.6) overnight at 4°C, and blocked with 5% SM-PBST. The samples used for probing consisted of NI rat serum at 1:200 (n = 3) or neat ASC-probes from infected rat skin LN (n = 5); lung LN (n = 5); liver LN (n = 5); and spleen (n = 4). Duplicate samples were incubated in the wells for 2 h at 37°C. After washing anti-rat Ig (H + L):HRP (1:5000) was incubated for 1 h at 37°C. TMB solution (Life Technologies) was added to each well, followed by 2 M H_2_SO_4_, and the optical density (OD) was read at 450 nm. Statistical differences in binding of ASC-probes to Sj-L6L-1 were assessed using a Kruskall-Wallis one-way analysis of variance.

## Results

### Protein array screening with ASC-probes for antigen identification

Screening of the 232 schistosome protein microarray with antibodies obtained from lymph nodes (ASC-probes) of *S. japonicum* challenged rats resulted in significant recognition of 11 antigens (Table 1). Nine of these were recognised by the lung ASC-probes, three by the skin ASC-probes and one (tetraspanin-2; TSP-2) was recognised by both skin and lung ASC-probes. Only 3 antigens were consistently recognised by all four of the lung ASC-probes; these were the novel hypothetical protein AY815838, and the known vaccine candidates TSP-2 and 21.7 kDa antigen (Sj21.7). Other known molecules that were recognised by the ASC-probes included 22.6 kDa antigen (Sj22.6), *S. mansoni* filamin, dynein light chain 1 (DLC1), *S. mansoni* tetraspanin-3, and the 29 kDa antigen (Sj29). Because the amount of protein in each spot is not standardised due to the cell-free expression system used [29,30], it should be noted, however, that the degree of antibody binding to proteins on the array does not necessarily indicate antigenicity. Therefore, even weakly recognised antigens, such as Ly-6-like protein 1 (Sj-L6L-1), which was only recognised by one skin and lung ASC-probe sample, may also be considered as relevant antigenic targets.

**Table 1:** ***S. japonicum *** **protein array antigen recognition by rat ASC-probes**

		Recognition* by ASC-probes							
**Name**	**Accession no.**	**Skin (No.)**		**Lung (No.)**		**Novel**^**∆**^	**Expression profile#**	**SignalP†**	**TM∞**
Tetraspanin-2 (SjTSP2)	EF553319.1	++	(3)	+++	(4)	No	S	No	Yes
**Hypothetical protein**	**AY815838**	**+**	**(1)**	**+++**	**(4)**	**Yes**	**S**	**Yes**	**Yes**
22.6 kDa antigen (Sj22.6)	L08198	-	(0)	+++	(3)	No	S, A	No	Yes
**Calponin-like**	**AY813467**	**-**	**(0)**	**++**	**(3)**	**Yes**	**S**	**No**	**No**
Filamin	XM_002571418	-	(0)	+++	(2)	No	C, S, A, Sp	No	Yes
21.7 kDa antigen (Sj21.7)	AF048759	+	(1)	++	(4)	No	S,A	No	No
Dynein light chain	AF072327.1	-	(0)	++	(2)	No	E, S	No	No
Zinc finger protein	AY223099	++	(1)	+	(2)	Yes	E, A	No	No
**Ly6-like (Sj-L6L-1)**	**AY816003**	**+**	**(1)**	**++**	**(1)**	**Yes**	**S**	**Yes**	**Yes**
Tetraspanin-3 SmTSP3 (*S. mansoni*)	CABG01000023	+	(3)	-	(0)	Yes	^	No	Yes
29 kDa antigen (Sj29)	DQ873812.1	+	(3)	+	(1)	No	S, A	Yes	Yes

*Recognition level score: the average signal intensity of the individual(s) which were greater than the mean + 2SD of control serum. High (+++) >10000 SI; Moderate (++) 500–9999 SI; Low (+) 0–499 SI; Negative (-) 0 SI. Number of ASC-probe samples (out of four) that recognised each antigen is indicated in parentheses. ^**∆**^Novel: defined as not having been tested as a vaccine candidate against *S. japonicum* and limited characterisation. #Expression profile: The stage(s) with the highest expression is indicated, based on EST Profile Viewer, UniGene, NCBI: cercariae (C), schistosomula (S), adult worm (A), egg (E), sporocyst (Sp). †Signal peptide determined by SignalP -4.1 (http://www.cbs.dtu.dk/services/SignalP/). ∞Transmembrane prediction by TMpred (http://www.ch.embnet.org/software/TMPRED_form.html) ^Data on sequence not present in UniGene. Three antigens possessing ideal characteristics as novel vaccine candidates are highlighted in bold.

To select antigens for further characterisation, each of the 11 sequences were investigated as to its novelty, its potential for up-regulated larval expression, and predicted exposure to the host’s immune system (indicated by the presence of a signal peptide and/or transmembrane domain). Five of the eleven proteins identified were novel and recognised by lung ASC probes, and three of these had indications that they were highly expressed in the schistosomula stage. Two of the novel antigens, AY815838 and Sj-L6L-1, were predicted to have signal peptides and transmembrane domains. The third novel antigen identified, calponin-like protein, had no predicted host-exposed features.

The AY815838 protein is currently unknown, but has limited amino acid sequence identity to two surface antigens of *S. mansoni*: Sm25 (accession #AAA29943; 34% identity) and Sm13 (accession #AAC25419.1; 30% identity). Sj-L6L-1 has significant homology to a *S. mansoni* antigen that was briefly investigated in a DNA vaccine study [31] and recently designated Sm-CD59.2 [32]. Finally calponin-like protein has a predicted size of 27 kDa and has some homology with a 38 kDa *S. japonicum* calponin homologue (accession #AAD11976; 63% identity) previously investigated [33].

### Developmental expression analysis of novel antigens

**Figure 1:**
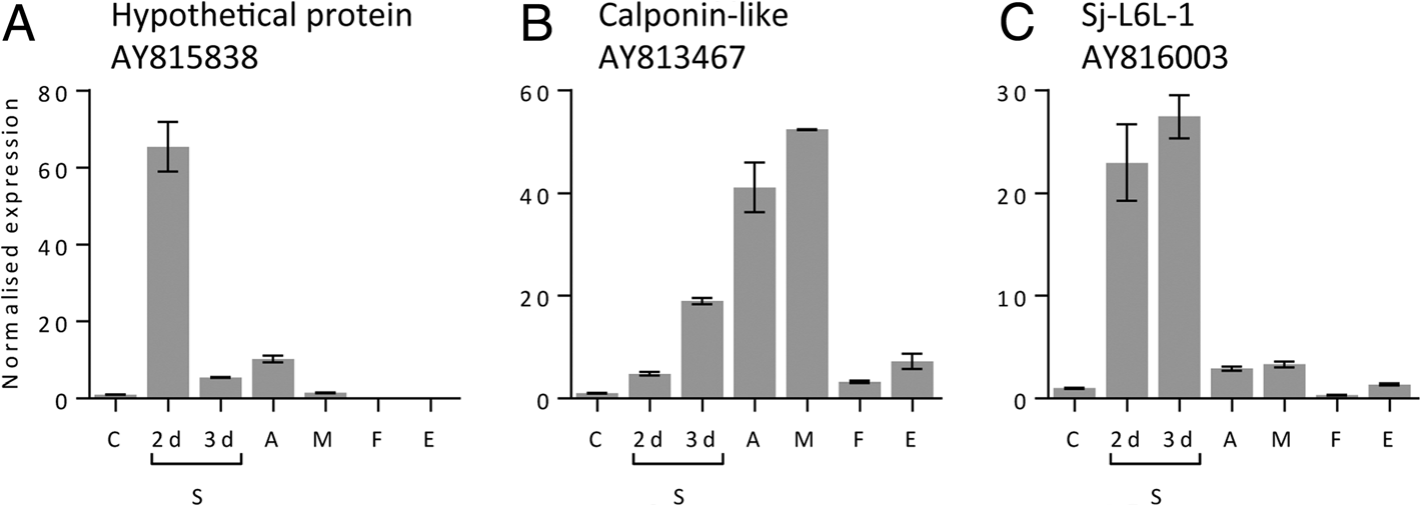
**Developmental expression of novel *S. japonicum *antigens recognised by lymph node-derived ASC-probes.** Expression levels of AY815838 **(A)**, calponin-like protein **(B)** and Sj-L6L-1 **(C)** in developmental stages of *S. japonicum* were determined by quantitative real-time PCR. Stages examined were cercariae (C), 2 day- (2d) and 3 day-old (3d) schistosomula (S), adult pairs (A), adult males (M), adult females (F) and eggs (E). Normalized fold expression of the genes relative to the expression in cercariae is presented, and bars represent standard error of the mean.

The developmental expression of the three novel antigen transcripts was investigated by qPCR (Figure 1). The AY815838 transcript was most highly expressed in the 2 day schistosomula, elevated to 65 times the cercarial expression, which then reduced to 5 and 10 times cercarial expression levels in 3-day schistosomula and adult pairs, respectively. The calponin-like protein showed a steady increase in expression throughout the development of the intravascular stages, peaking in the adult worms at 41 times the cercarial expression. Finally, Sj-L6L-1 was very highly expressed in the developing schistosomula; the 2-day *in vitro* cultured and the 3-day *in vivo* lung-stage larvae had 23 and 27 times the cercarial expression respectively, whereas adult males had just a 3-fold -increase in transcript level. For all three genes, expression in adult worms was predominantly, or restricted to, the males.

These data suggested AY815838 and Sj-L6L-1 were promising novel targets with predominant larval expression. The larval expression of Sj-L6L-1 was particularly convincing since there was strong agreement in transcript levels in samples from both *in vitro* and *in vivo*-generated larvae (2- and 3-day schistosomula, respectively). AY815838 was identified in a previous screening of the microarray and is being currently investigated (P. Driguez and D. McManus, personal communication); therefore Sj-L6L-1 was selected for further detailed characterisation.

### Sequence analysis of Sj-L6L-1

**Figure 2:**
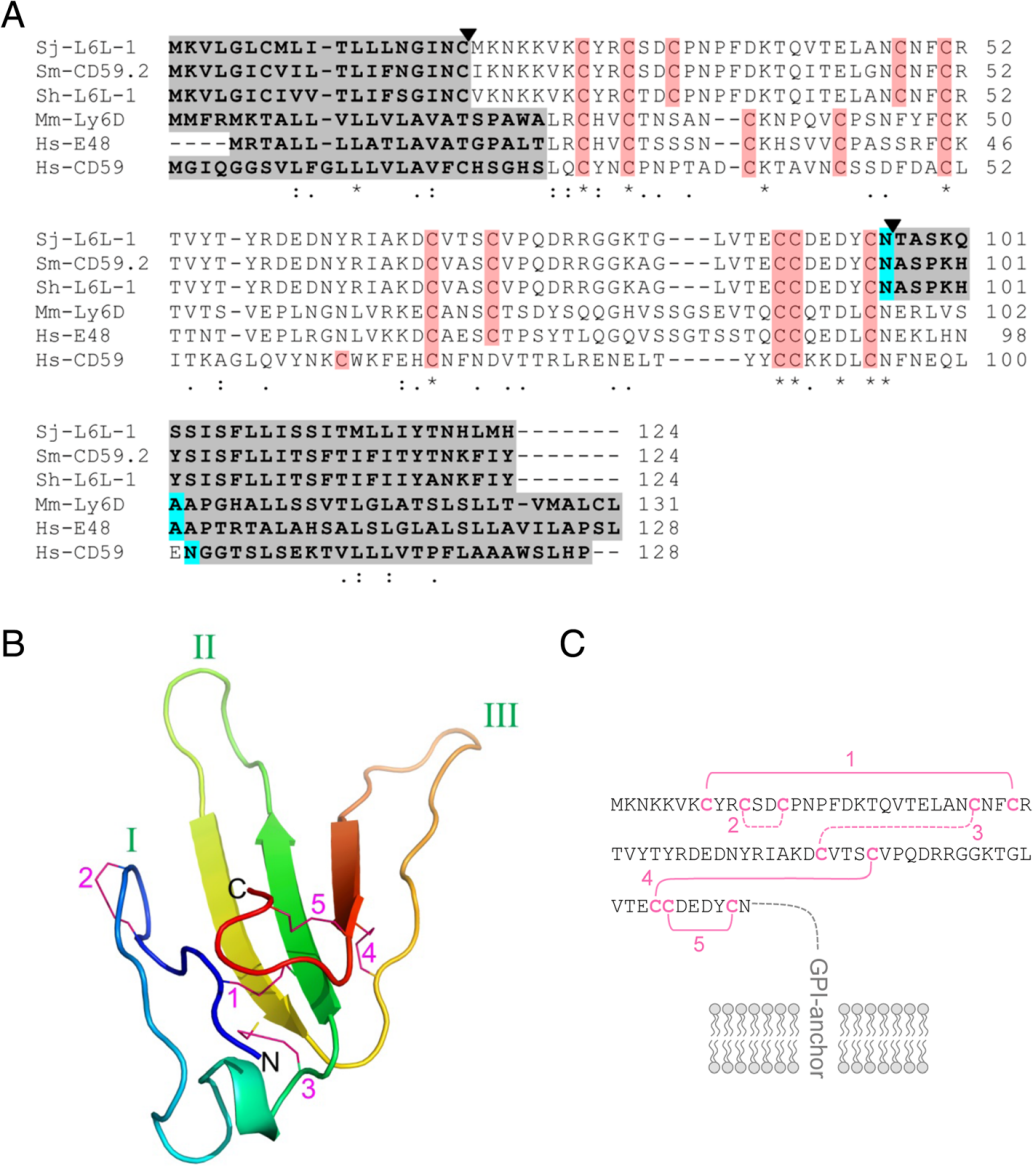
**Sequence and predicted structure of mature Sj-L6L-1. A**: The Sj-L6L-1 sequence aligned with the highly similar orthologous sequences from *S. mansoni* (Sm-CD59.2) and *S. haematobium* (Sh-L6L-1) and compared with Ly-6 family proteins from mice (Mm-Ly-6D) and humans (Hs-E48 and Hs-CD59). The N-terminal signal peptide and the C-terminal propeptide are highlighted in grey, while structurally important cysteine residues are highlighted in red. The predicted omega site (GPI-anchor site) is highlighted in blue. The predicted mature Sj-L6L-1 sequence is indicated within the black triangles. **B**: The predicted structure of mature Sj-L6L-1 was modelled based on a Phyre2 structure homology search, revealing a structure with the typical three-finger fold common to Ly-6 family proteins. Each finger is labelled with green roman numerals. **C**: The mature Sj-L6L-1 amino acid sequence has the predicted disulfide bonding pattern, also characteristic of this family, including the GPI-anchor at the C-terminus. The disulfide bonds are shown and numbered in magenta, with those predicted by the *in silico* analysis depicted by solid lines and those manually inferred by dotted lines.

Database searching revealed Sj-L6L-1 has similarity with the Lymphocyte Antigen 6 (Ly-6) family of proteins, present in many eukaryotic species (Figure 2). The membrane-anchored members of this family share several features: a predicted N-terminal signal peptide, a glycosylphosphatidylinositol (GPI)-anchor omega site where the C-terminal propeptide is cleaved off in the mature protein, and the Ly-6/uPAR (LU) protein domain characterised by 8–10 conserved cysteine residues [34].

Highly related orthologues to Sj-L6L-1 were found in *S. mansoni* (SmCD59.2; accession number XP_002570561) and *S. haematobium* (Sh-L6L-1; B_00489), which have 78 and 79*%* amino acid identity, respectively. All three have most of the classical Ly-6 family features: predicted signal peptides, GPI-anchor omega site, and ten cysteines in the mature region (eight of these align with conserved mammalian residues but two are in differing positions) (Figure 2A). However, domain searching using the amino acid sequence did not predict the presence of an LU domain in these schistosome orthologues, most likely due to the altered positions of the 3rd and 4th cysteines (see Figure 2A). These altered cysteines were common in all trematode orthologues examined (Figure 2A and data not shown). The *S. mansoni* orthologue, SmCD59.2, was recently described along with other members of its family by Farias et al. [32]. This family was referred to as CD59-like proteins because they have some similarity with the mammalian Ly-6 family member, CD59. However, since the schistosome proteins more closely resemble the family of Ly-6 proteins rather than one particular member, we propose to describe them as ‘Ly-6-like’ proteins. *S. japonicum* also has several orthologous sequences to Sj-L6L-1, although the closest Sj-L6L-2 (accession number AAW26563.1), has only 38% identity.

The full pre-protein sequence of Sj-L6L-1 is predicted to be 14.1 kDa, while the mature protein after the signal peptide and propeptide are cleaved is estimated to be 8.9 kDa. However, the addition of the GPI anchor is estimated to add a similar amount to the cleaved propeptide [35], so the native form would be approximately 12 kDa.

Other parasitic trematode sequences with low identity (31-34*%*) to Sj-L6L-1 were identified in *F. hepatica* (Fh_Contig6273), *F. gigantica* (Contig25430), *C.sinensis* (CS1_c757) and *O. viverrini* (OV1_c8524). The closest mammalian orthologues are the mouse Ly-6D (also known as ThB; 28*%* identity; accession # EDL29445), the human E48 protein (32*%* identity; accession # CAA73189). Human CD59 in contrast has 25*%* identity (accession # CAG46523).

Despite not having a traditional LU domain by sequence searching, structural homology searching of the mature Sj-L6L-1 sequence predicted structural similarity with Ly-6 proteins. The highest scoring template following the Phyre2 server search was the Ly-6 protein Lynx1, with 98.3% confidence. As shown in Figure 2B, *in silico* modelling predicted a three-fingered structure which is common to Ly-6-like proteins [36]. This structure was predicted to contain 3 disulfide bonds, numbered 1, 4 and 5 in Figure 2B and C. Disulfide bonds 2 and 3 depicted in Figure 2 were manually added since this is the same pattern seen in other Ly-6 proteins such as human Lynx1 [37] and CD59 [38,39], and because the residues are in close proximity in the model (Figure 2B).

### Recombinant Sj-L6L-1 is in the native antigenic conformation

**Figure 3:**
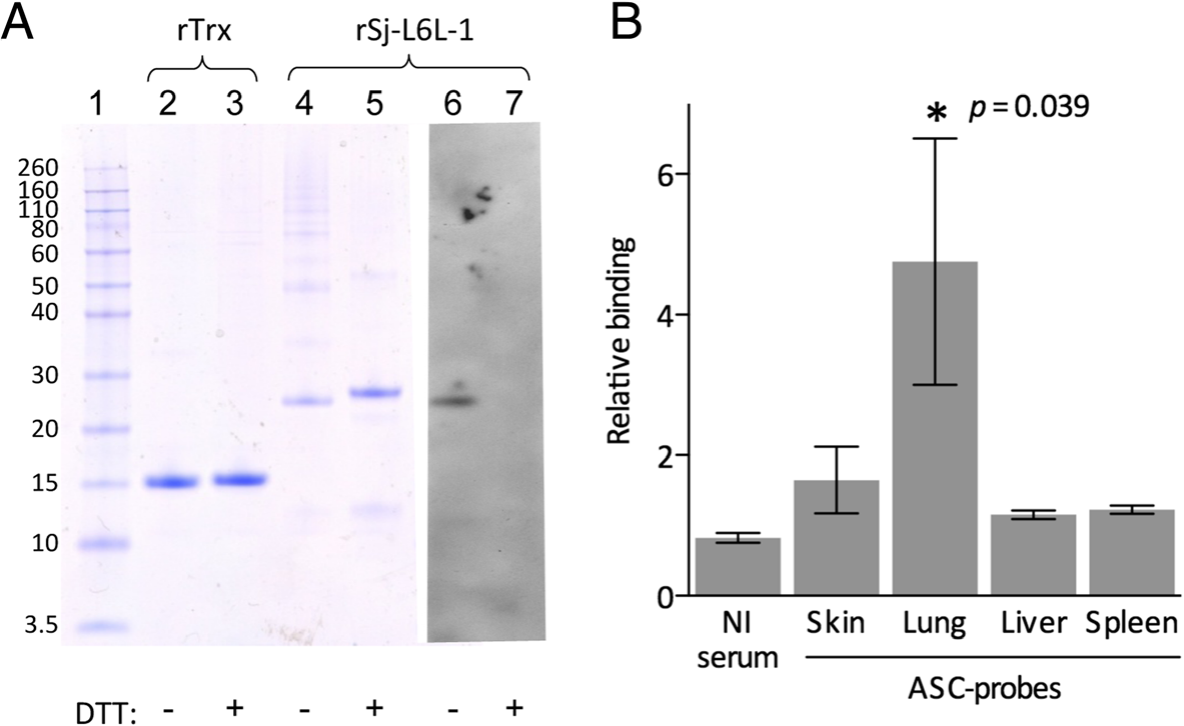
**Recombinant Sj-L6L-1 (rSj-L6L-1) contains conformational epitopes. A**: The purified fusion protein of rSj-L6L-1 with thioredoxin (Trx), or rTrx alone, were analysed by SDS-PAGE under non-reducing conditions (lanes 2, 4, 6) or reducing conditions with dithiothreitol (DTT; lanes 3, 5, 7). DTT had no effect on rTrx but slightly increased the size of rSj-L6L-1. Reduced and non-reduced rSj-L6L-1 was transferred to a nitrocellulose membrane and probed with lung-LN ASC-probes (lanes 6–7). **B**: rSj-L6L-1 ELISA with rat ASC-probes obtained from skin-, lung- or liver-LN or spleen compared with non-infected (NI) rat serum. Relative binding to rSj-L6L-1 is the ratio of the rSj-L6L-1 optical density to that of the fusion partner, rTrx, alone. Bars represent the mean ± standard error.

Recombinant Sj-L6L-1 was produced fused to thioredoxin (Trx) as a solubility tag, and induction with arabinose caused a significant expression of a soluble protein band at approximately 27 kDa as observed by reducing SDS-PAGE (Figure 3). This was purified by the 6His tag using a nickel column. The protein band was excised and the sequence confirmed by LC-MS/MS using a HCT ULTRA ion trap mass spectrometer (Bruker Daltonics; Monash Biomedical Proteomics Facility). Under non-reducing conditions the protein had a molecular weight of 25 kDa (Figure 3A, lane 4). This reduction in size was likely due to a change in the Sj-L6L-1 portion of the fusion protein, since reducing or non-reducing conditions had no effect on the size of rTrx which remained at 16 kDa (lanes 2 and 3). Finally, immunoblotting revealed rSj-L6L-1 was recognised by the rat lung ASC-probes from infected rats, but only in the non-reduced form (Figure 3A, lane 6). This indicated that the recombinant protein shares conformational epitopes with the native protein, and these are abolished by treating with reducing agent, presumably by disrupting the disulfide bonds between the structurally-important cysteines.It was also noted that there was a ladder effect with rSj-L6L-1 in the non-reduced sample (Figure 3A, lane 4). These corresponded to multimers of the protein, visible at 50 kDa (dimer) and 75 kDa (trimer) and then increasing masses that were not distinguishable. In the reduced sample (lane 5) only an additional 55 kDa band was present, likely to be a dimer of the reduced form. These bands also reacted with an anti-His tag antibody (data not shown), indicating that they are aggregates of rSj-L6L-1, but none of the multimers were recognised by the lung ASC-probes compared to the 25 kDa monomer.

### Recombinant Sj-L6L-1 is recognised by the local lung antibody response in rats

Since lung ASC-probes were found to recognise non-reduced rSj-L6L-1 (Figure 3A), an ELISA was used to investigate the binding of the different rat ASC-probe samples to rSj-L6L-1 (Figure 3B). To account for some background binding to the fusion tag alone, the data are presented as relative binding (RB) of rSj-L6L-1 to rTrx, where RB > 1 means the sample bound to rSj-L6L-1 greater than the rTrx control. Figure 3B illustrates that the non-infected (NI) rat sera had no recognition of rSj-L6L-1 with a mean of 0.8 RB. The only sample type to have a statistically significant recognition of rSj-L6L-1 was the lung ASC-probes, with a mean of 4.8 RB (*p* = 0.039). The skin ASC-probes had some slight recognition of rSj-L6L-1 at 1.6 RB, although this was not significantly higher than the other groups.

### Native Sj-L6L-1 is present in tegument extract

**Figure 4:**
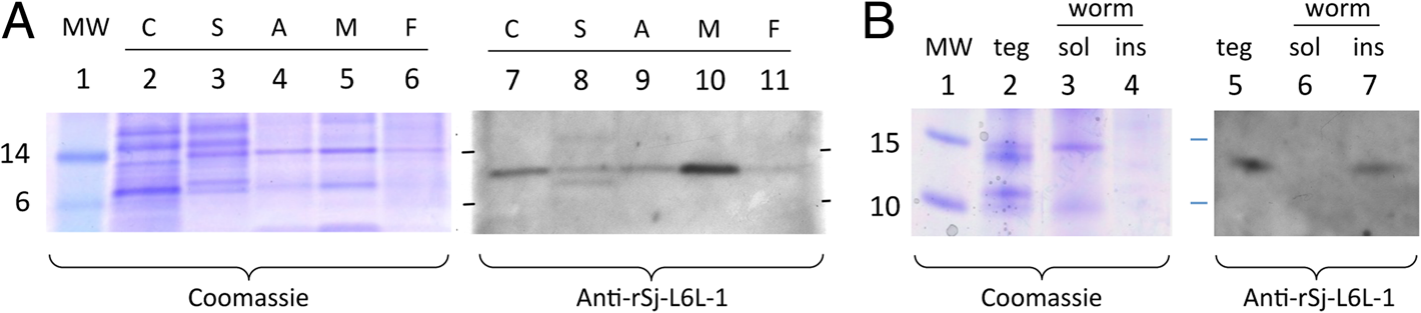
**Detection of native Sj-L6L-1 in *S. japonicum *extracts. A**: Protein extracts from *S. japonicum* cercariae (C), schistosomula (S), adult worm pairs (A), or separated male (M) or female (F) worms were analysed by SDS-PAGE and stained with coomassie (lanes 2–6) or subjected to immunoblotting (lanes 7–11) with antisera to rSj-L6L-1. Molecular weight (MW) markers are shown in lane 1. **B**: Adult worms were fractionated into tegument (teg), aqueous-soluble (sol) and -insoluble (ins) worm fractions, and analysed by immunoblotting with antisera to rSj-L6L-1.

Schistosome extracts probed with antiserum to rSj-L6L-1 recognised a band of approximately 12 kDa in all developmental stages examined (Figure 4A), with an additional band of approximately 10 kDa observed only in the schistosomula. Neither of these were recognised by antiserum to rTrx (data not shown). The 12 kDa Sj-L6L-1 band was similar in intensity comparing schistosomula and adult worm pairs, and it was clear that the protein is more abundant in male worms than females. Due to the scarcity of larval material and the difficulty in removing the larval tegument [40], tegument extracts were generated from adult worms only. The same 12 kDa band was seen in the worm tegument and insoluble fractions (Figure 4B), but not in the aqueous-soluble fraction. This indicates rSj-L6L-1 is located in the tegument and is membrane-associated.

## Discussion

In this study, a novel post-genomic technique, a protein microarray, was employed to identify schistosome vaccine candidates. To focus on the identification of antigens important in the vulnerable larval stages, rat lymph node antibody samples (ASC-probes) generated previously from the sites of larval infection [17], the skin and lungs, were used to screen the arrays. The combination of these two ‘immunomic’ technologies – the protein microarray, providing multiple antigen binding data, and the ASC-probe samples, which provide tissue-specific reactivity, resulted in a list of targets which may contribute to a vaccine targeting the migrating larvae [18]. Several novel and also known vaccine candidates were recognised; one of these, named Sj-L6L-1, was further characterised and has several features suggesting it is a promising vaccine candidate relevant to the developing larvae.

The known targets included TSP-2, a tegumental antigen for which the homologue in *S. mansoni* is currently under further development as a vaccine candidate [2]. The present study suggests that TSP-2 is exposed to the host’s immune system in both the skin and lung sites during rat *S. japonicum* infection, since it was strongly recognised by skin and lung ASC-probes. Other known vaccine candidates recognised by the lung ASC-probes were: Sj22.6 and Sj21.7, both tegument-associated proteins from the TAL protein family [41]; *S. mansoni* filamin, a large structural protein shown to confer some protection in a mouse model [42]; and DLC1 which has been found associated with the worm surface [43,44]. In addition, two further molecules, *S. mansoni* TSP-3 and Sj29, were recognised by the skin ASC-probes, indicating that they are antigenic predominantly during skin invasion. These are both purported to be surface molecules and Sj29 has been investigated as a vaccine candidate [45]. Gobert et al. [46] found that in *S. mansoni,* the genes encoding both proteins had significantly up-regulated transcript expression after transformation from cercariae to larvae, and notably Sm29 (the homologue of Sj29) had the highest expression in the 3 hour schistosomulum, the stage which develops shortly after skin penetration.

A major criterion used in the present study to select novel antigens for further characterisation was increased larval expression. Initially, the NCBI EST database was used as a preliminary measure of developmental expression. This indicated that only one of the four novel targets was not up-regulated in schistosomula, the zinc finger protein (AY223099). This antigen also lacked a signal peptide and transmembrane domain, and is likely to be an intracellular protein and therefore may only be exposed to the host’s immune system during parasite damage. The developmental expression of the remaining three antigens was confirmed by qPCR, and both AY815838 and Sj-L6L-1 showed high larval gene expression relative to cercariae. AY815838 was dramatically up-regulated in the 2 day *in vitro-*cultured, but not the 3 day *in vivo* lung-isolated, schistosomula. This high expression in the *in vitro* larvae could indicate an early high expression that is then reduced after 3 days, or could be an artefact of *in vitro* culture. Sj-L6L-1, however, showed a more consistent larval expression, with similar up-regulated levels in the 2 day and 3 day larvae compared with other stages.

Based on these analyses, Sj-L6L-1 was selected for further characterisation and was produced in recombinant form. The *E. coli*-expressed rSj-L6L-1 fusion protein was recognised by the lung ASC-probes indicating that it was at least partly in the correct antigenic conformation. By treating with reducing agent, the protein structure was altered sufficiently to ablate recognition by these antibodies, indicating that the schistosomiasis-induced antibodies recognised only conformational epitopes on the recombinant protein The disulfide bonds in Ly-6 proteins are known to be important for their structural conformation, stabilising the typical three-finger motif; for example, when CD59 is treated with reducing agent it loses its ability to inhibit the complement system [39].

The Sj-L6L-1 protein was identified in all life-stages examined. No increase in protein expression was seen in the schistosomula despite the significant increase in transcription. However, an additional band was recognised in the schistosomula and this could represent either an immature form of the protein or another variation unique to this developmental stage. The Sj-L6L-1 protein was also detected in the tegument extract from adult *S. japonicum* worms, and the same band was found in the insoluble fraction from the ‘denuded’ adult worms. This indicates that Sj-L6L-1 is at least associated with the outer tegument, and is highly likely to be on the external surface as is the case for its homologue in *S. mansoni*[47]. It also suggests that the protein is attached to the plasma membrane, since it was only detected in the insoluble fraction; hence, it is unlikely to be secreted like some Ly-6 proteins.

Another important observation was that local antibodies obtained from rat lymph nodes draining the lung, the site of larval killing, were specific for rSj-L6L-1. No specific antibody was evident against the protein in lymph node samples obtained from the liver, where more mature worms reside, despite the fact that male adults do produce the protein. This suggests that Sj-L6L-1 is uniquely exposed during larval development and not in adult worms. It is possible that this occurs after the transformation of the schistosomula from cercariae, when the larvae rapidly synthesise the new tegument [48] and before they acquire host proteins which mask their own antigens [9].

While Sj-L6L-1 is novel for *S. japonicum*, the closest homologue in *S. mansoni* (SmCD59.2) and its family have been recently characterised [32]. Previously, two of the members of this family were identified in the adult *S. mansoni* worm tegument using proteomics [47]. Farias et al. [31] analysing the *S. mansoni* transcriptome for genes up-regulated from the cercariae to the schistosomula, identified SmCD59.2 (which they refer to as ‘dif 5’) and performed a limited DNA vaccine trial which resulted in a slight (but non-significant) reduction (22%) in worm burden.

The Ly-6 family of proteins was originally described in mice, but Ly-6-like proteins have since been found in many animal species from *C. elegans* to humans and comprise the Ly-6 super gene family [49]. These are broadly grouped together based on the presence of 8–10 conserved cysteines which comprise the LU domain [34]. These conserved cysteines create 4–5 disulfide bonds resulting in a three-finger structure, a motif also common to the related snake venom toxins [36]. Since Sj-L6L-1 is related to this family by containing most of these features, it is referred to here as Ly-6-like and part of the Ly-6 super family. The Ly-6 family members and Ly-6-like proteins appear to have extremely diverse roles, although their precise functions are as yet unclear [50]. This family of proteins also exhibit limited sequence identity between members [49], which makes assigning a putative function to Sj-L6L-1 difficult. They are generally thought to participate in development, cell adhesion, and cell signalling, although how the latter occurs is still unknown [49].

## Conclusions

In summary, a novel protein microarray was combined with a tissue-specific antibody source to investigate the immunome of migrating *S. japonicum* larvae and to identify novel antigens expressed by the schistosomula stage, which is generally considered the likely target of an antischistosome vaccine. Several novel and known proteins were found to be antigenic in the regions of larval migration and could form part of a multivalent vaccine specifically targeting the schistosomula. Although these targets were identified using rat samples, their vaccine efficacy should ideally be tested in a final host such as the water buffalo to account for differences in immune mechanisms between species [51]. Of the identified molecules, a novel *S. japonicum* protein, Sj-L6L-1, was characterised and found to be antigenic in the larval stage and present, but not antigenic, in the worm tegument, and may provide a valuable vaccine candidate against schistosomiasis.

## Competing interests

The authors declare that they have no competing interests.

## Author’s contributions

PD probed the microarrays, HM & PD analysed the microarray data and isolated parasite samples, HM synthesised and characterised Sj-L6L-1, and drafted the manuscript. HM, PD, EM, DP and DPM interpreted results and edited, drafted and approved the final manuscript.

## References

[B1] McManus DP Loukas A Current status of vaccines for schistosomiasis Clin Microbiol Rev 2008 21 1 225 242 18202444 10.1128/CMR.00046-07 PMC2223839

[B2] Hotez PJ Bethony JM Diemert DJ Pearson M Loukas A Developing vaccines to combat hookworm infection and intestinal schistosomiasis Nat Rev Microbiol 2010 8 11 814 826 20948553 10.1038/nrmicro2438

[B3] Bergquist R Utzinger J McManus DP Trick or treat: the role of vaccines in integrated schistosomiasis control PLoS Negl Trop Dis 2008 2 6 e244 18575619 10.1371/journal.pntd.0000244 PMC2430529

[B4] Bickle QD Radiation-attenuated schistosome vaccination - a brief historical perspective Parasitology 2009 136 12 1621 1632 19327194 10.1017/S0031182009005848

[B5] Wilson RA Coulson PS Schistosome vaccines: a critical appraisal Mem Inst Oswaldo Cruz 2006 101 Suppl 1 13 20 17308743 10.1590/s0074-02762006000900004

[B6] Pearce EJ MacDonald AS The immunobiology of schistosomiasis Nat Rev Immunol 2002 2 7 499 511 12094224 10.1038/nri843

[B7] McManus DP Gray DJ Li Y Feng Z Williams GM Stewart D Rey-Ladino J Ross AG Schistosomiasis in the People’s Republic of China: the era of the three gorges dam Clin Microbiol Rev 2010 23 2 442 466 20375361 10.1128/CMR.00044-09 PMC2863366

[B8] Gryseels B Polman K Clerinx J Kestens L Human schistosomiasis Lancet 2006 368 9541 1106 1118 16997665 10.1016/S0140-6736(06)69440-3

[B9] Skelly PJ Wilson A Making sense of the schistosome surface Adv Parasitol 2006 63 185 284 17134654 10.1016/S0065-308X(06)63003-0

[B10] Harris AR Russell RJ Charters AD A review of schistosomiasis in immigrants in Western Australia, demonstrating the unusual longevity of Schistosoma mansoni Trans R Soc Trop Med Hyg 1984 78 3 385 388 6464135 10.1016/0035-9203(84)90129-9

[B11] McWilliam HEG Driguez P Piedrafita D McManus DP Meeusen ENT Novel immunomic technologies for schistosome vaccine development Parasite Immunol 2012 34 5 276 284 22486551 10.1111/j.1365-3024.2011.01330.x

[B12] Zhou Y Zheng H Liu F Hu W Wang ZQ Gang L Ren S The schistosoma japonicum genome reveals features of host-parasite interplay Nature 2009 460 7253 345 351 19606140 10.1038/nature08140 PMC3747554

[B13] Berriman M Haas BJ LoVerde PT Wilson RA Dillon GP Cerqueira GC Mashiyama ST Al-Lazikani B Andrade LF Ashton PD Aslett MA Bartholomeu DC Blandin G Caffrey CR Coghlan A Coulson R Day TA Delcher A DeMarco R Djikeng A Eyre T Gamble JA Ghedin E Gu Y Hertz-Fowler C Hirai H Hirai Y Houston R Ivens A Johnston DA The genome of the blood fluke Schistosoma mansoni Nature 2009 460 7253 352 358 19606141 10.1038/nature08160 PMC2756445

[B14] Young ND Jex AR Li B Liu S Yang L Xiong Z Li Y Cantacessi C Hall RS Xu X Chen F Wu X Zerlotini A Oliveira G Hofmann A Zhang G Fang X Kang Y Campbell BE Loukas A Ranganathan S Rollinson D Rinaldi G Brindley PJ Yang H Wang J Gasser RB Whole-genome sequence of schistosoma haematobium Nat Genet 2012 44 2 221 225 22246508 10.1038/ng.1065

[B15] Walker A Insights into the functional biology of schistosomes Parasit Vectors 2011 4 1 203 22013990 10.1186/1756-3305-4-203 PMC3206467

[B16] Driguez P Doolan DL Loukas A Felgner PL McManus DP Schistosomiasis vaccine discovery using immunomics Parasit Vectors 2010 3 4 20181031 10.1186/1756-3305-3-4 PMC2837634

[B17] McWilliam HE Driguez P Piedrafita D Maupin KA Haab BB McManus DP Meeusen EN The developing schistosome worms elicit distinct immune responses in different tissue regions Immunol Cell Biol 2013 91 7 477 485 23856766 10.1038/icb.2013.33

[B18] Capron M Capron A Rats, mice and men - models for immune effector mechanisms against schistosomiasis Parasitol Today 1986 2 3 69 75 15462774 10.1016/0169-4758(86)90158-4

[B19] Hu Y Lu W Shen Y Xu Y Yuan Z Zhang C Wu J Ni Y Liu S Cao J Immune changes of schistosoma japonicum infections in various rodent disease models Exp Parasitol 2012 131 2 180 189 22521591 10.1016/j.exppara.2012.03.022

[B20] Han H Peng J Hong Y Zhang M Han Y Fu Z Shi Y Xu J Tao J Lin J Comparison of the differential expression miRNAs in wistar rats before and 10 days after S.Japonicum infection Parasit Vectors 2013 6 1 120 23617945 10.1186/1756-3305-6-120 PMC3640946

[B21] Huber W von Heydebreck A Sultmann H Poustka A Vingron M Variance stabilization applied to microarray data calibration and to the quantification of differential expression Bioinformatics 2002 18 Suppl 1 S96 S104 12169536 10.1093/bioinformatics/18.suppl_1.s96

[B22] Sundaresh S Doolan DL Hirst S Mu Y Unal B Davies DH Felgner PL Baldi P Identification of humoral immune responses in protein microarrays using DNA microarray data analysis techniques Bioinformatics 2006 22 14 1760 1766 16644788 10.1093/bioinformatics/btl162

[B23] Trieu A Kayala MA Burk C Molina DM Freilich DA Richie TL Baldi P Felgner PL Doolan DL Sterile protective immunity to malaria is associated with a panel of novel P. falciparum antigens Mol Cell Proteomics 2011 10 9 M111 007948 10.1074/mcp.M111.007948 PMC3186199 21628511

[B24] Gobert GN Moertel L Brindley PJ McManus DP Developmental gene expression profiles of the human pathogen schistosoma japonicum BMC Genomics 2009 10 128 19320991 10.1186/1471-2164-10-128 PMC2670322

[B25] Liu S Cai P Hou N Piao X Wang H Hung T Chen Q Genome-wide identification and characterization of a panel of house-keeping genes in schistosoma japonicum Mol Biochem Parasitol 2012 182 1–2 75 82 22245333 10.1016/j.molbiopara.2011.12.007

[B26] Kelley LA Sternberg MJ Protein structure prediction on the web: a case study using the phyre server Nat Protoc 2009 4 3 363 371 19247286 10.1038/nprot.2009.2

[B27] Roberts SM MacGregor AN Vojvodic M Wells E Crabtree JE Wilson RA Tegument surface membranes of adult schistosoma mansoni: development of a method for their isolation Mol Biochem Parasitol 1983 9 2 105 127 6669162 10.1016/0166-6851(83)90104-4

[B28] Mulvenna J Moertel L Jones MK Nawaratna S Lovas EM Gobert GN Colgrave M Jones A Loukas A McManus DP Exposed proteins of the schistosoma japonicum tegument Int J Parasitol 2010 40 5 543 554 19853607 10.1016/j.ijpara.2009.10.002

[B29] Doolan DL Plasmodium immunomics Int J Parasitol 2011 41 1 3 20 20816843 10.1016/j.ijpara.2010.08.002 PMC3005034

[B30] Davies DH Liang X Hernandez JE Randall A Hirst S Mu Y Romero KM Nguyen TT Kalantari-Dehaghi M Crotty S Baldi P Villarreal LP Felgner PL Profiling the humoral immune response to infection by using proteome microarrays: high-throughput vaccine and diagnostic antigen discovery Proc Natl Acad Sci U S A 2005 102 3 547 552 15647345 10.1073/pnas.0408782102 PMC545576

[B31] Farias LP Tararam CA Miyasato PA Nishiyama MY Jr Oliveira KC Kawano T Verjovski-Almeida S Leite LC Screening the schistosoma mansoni transcriptome for genes differentially expressed in the schistosomulum stage in search for vaccine candidates Parasitol Res 2011 108 1 123 135 20852890 10.1007/s00436-010-2045-1

[B32] Farias LP Krautz-Peterson G Tararam CA Araujo-Montoya BO Fraga TR Rofatto HK Silva-Jr FP Isaac L Da’dara AA Wilson RA Shoemaker CB Leite LCC On the three-finger protein domain fold and CD59-like proteins in *Schistosoma mansoni* PLoS Negl Trop Dis 2013 7 10 e2482 24205416 10.1371/journal.pntd.0002482 PMC3812095

[B33] Yang W Zheng YZ Jones MK McManus DP Molecular characterization of a calponin-like protein from schistosoma japonicum Mol Biochem Parasitol 1999 98 2 225 237 10080391 10.1016/s0166-6851(98)00171-6

[B34] Stroncek DF Caruccio L Bettinotti M CD177: a member of the Ly-6 gene superfamily involved with neutrophil proliferation and polycythemia Vera J Transl Med 2004 2 1 8 15050027 10.1186/1479-5876-2-8 PMC411062

[B35] Patel BN David S A novel glycosylphosphatidylinositol-anchored form of ceruloplasmin is expressed by mammalian astrocytes J Biol Chem 1997 272 32 20185 20190 9242695 10.1074/jbc.272.32.20185

[B36] Fry BG Wuster W Kini RM Brusic V Khan A Venkataraman D Rooney AP Molecular evolution and phylogeny of elapid snake venom three-finger toxins J Mol Evol 2003 57 1 110 129 12962311 10.1007/s00239-003-2461-2

[B37] Miwa JM Ibanez-Tallon I Crabtree GW Sanchez R Sali A Role LW Heintz N Lynx1, an endogenous toxin-like modulator of nicotinic acetylcholine receptors in the mammalian CNS Neuron 1999 23 1 105 114 10402197 10.1016/s0896-6273(00)80757-6

[B38] Sugita Y Nakano Y Oda E Noda K Tobe T Miura NH Tomita M Determination of carboxyl-terminal residue and disulfide bonds of MACIF (CD59), a glycosyl-phosphatidylinositol-anchored membrane protein J Biochem 1993 114 4 473 477 8276756 10.1093/oxfordjournals.jbchem.a124202

[B39] Morgan BP Tomlinson S Lambris JD, Morikis D Structure-Function Relationships in CD59 Structural Biology of the Complement System 2005 Boca Raton: Taylor & Francis 251 264

[B40] Wilson RA Proteomics at the schistosome-mammalian host interface: any prospects for diagnostics or vaccines? Parasitology 2012 139 9 1178 1194 22717150 10.1017/S0031182012000339

[B41] Fitzsimmons CM Jones FM Stearn A Chalmers IW Hoffmann KF Wawrzyniak J Wilson S Kabatereine NB Dunne DW The Schistosoma mansoni tegumental-allergen-like (TAL) protein family: influence of developmental expression on human IgE responses PLoS Negl Trop Dis 2012 6 4 e1593 22509417 10.1371/journal.pntd.0001593 PMC3317908

[B42] Cook RM Carvalho-Queiroz C Wilding G LoVerde PT Nucleic acid vaccination with schistosoma mansoni antioxidant enzyme cytosolic superoxide dismutase and the structural protein filamin confers protection against the adult worm stage Infect Immun 2004 72 10 6112 6124 15385516 10.1128/IAI.72.10.6112-6124.2004 PMC517585

[B43] Yang W Jones MK Fan J Hughes-Stamm SR McManus DP Characterisation of a family of schistosoma japonicum proteins related to dynein light chains Biochim Biophys Acta 1999 1432 1 13 26 10366724 10.1016/s0167-4838(99)00089-8

[B44] Zhang LH McManus DP Sunderland P Lu XM Ye JJ Loukas A Jones MK The cellular distribution and stage-specific expression of two dynein light chains from the human blood fluke schistosoma japonicum Int J Biochem Cell Biol 2005 37 7 1511 1524 15833281 10.1016/j.biocel.2005.01.015

[B45] Balloul JM Grzych JM Pierce RJ Capron A A purified 28,000 Dalton protein from schistosoma mansoni adult worms protects rats and mice against experimental schistosomiasis J Immunol 1987 138 10 3448 3453 3106483

[B46] Gobert GN Tran MH Moertel L Mulvenna J Jones MK McManus DP Loukas A Transcriptional changes in schistosoma mansoni during early schistosomula development and in the presence of erythrocytes PLoS Negl Trop Dis 2010 4 2 e600 20161728 10.1371/journal.pntd.0000600 PMC2817720

[B47] Castro-Borges W Dowle A Curwen RS Thomas-Oates J Wilson RA Enzymatic shaving of the tegument surface of live schistosomes for proteomic analysis: a rational approach to select vaccine candidates PLoS Negl Trop Dis 2011 5 3 e993 21468311 10.1371/journal.pntd.0000993 PMC3066142

[B48] Hockley DJ McLaren DJ Schistosoma mansoni: changes in the outer membrane of the tegument during development from cercaria to adult worm Int J Parasitol 1973 3 1 13 20 4687430 10.1016/0020-7519(73)90004-0

[B49] Bamezai A Mouse Ly-6 proteins and their extended family: markers of cell differentiation and regulators of cell signaling Arch Immunol Ther Exp (Warsz) 2004 52 4 255 266 15467490

[B50] Mallya M Campbell RD Aguado B Characterization of the five novel Ly-6 superfamily members encoded in the MHC, and detection of cells expressing their potential ligands Protein Sci 2006 15 10 2244 2256 17008713 10.1110/ps.062242606 PMC2242401

[B51] McWilliam HEG Piedrafita D Li Y Zheng M He Y Yu X McManus DP Meeusen ENT Local immune responses of the Chinese water buffalo, *bubalus bubalis*, against *schistosoma japonicum* larvae: crucial insights for vaccine design PLoS Negl Trop Dis 2013 7 9 e2460 24086786 10.1371/journal.pntd.0002460 PMC3784499

